# Transformation of *Lactuca sativa* L. with *rol C* gene results in increased antioxidant potential and enhanced analgesic, anti-inflammatory and antidepressant activities in vivo

**DOI:** 10.1007/s13205-016-0533-4

**Published:** 2016-10-05

**Authors:** Hammad Ismail, Erum Dilshad, Mohammad Tahir Waheed, Moniba Sajid, Waqas Khan Kayani, Bushra Mirza

**Affiliations:** 1 Department of Biochemistry and Molecular Biology, University of Gujrat, Gujrat, 50700 Pakistan; 2 Department of Bioinformatics and Biosciences, Capital University of Science and Technology, Islamabad, Pakistan; 3 Department of Biochemistry, Quaid-i-Azam University, Islamabad, 45320 Pakistan; 4 Department of Botany, The University of Poonch Rawalakot, Azad Kashmir, Pakistan

**Keywords:** *Agrobacterium tumefaciens*, Analgesic, Anti-inflammatory, Antidepressant, Antioxidants, *Lactuca sativa*, *rol* genes, Semiquantitative PCR

## Abstract

Lettuce is an important edible crop which possesses various medicinal properties. In this study *Lactuca sativa* L. (cv Grand Rapids) was transformed by *Agrobacterium*-mediated transformation with *rol C* gene. Transgene integration and expression was confirmed through PCR and semiquantitative RT-PCR. The transformed extracts were evaluated for their in vitro antioxidant and in vivo analgesic, anti-inflammatory and antidepressant activities in rats. The transformed plants showed 53–98 % increase in total phenolic and 45–58 % increase in total flavonoid contents compared with untransformed plants. Results of total reducing power and total antioxidant capacity exhibited 90–118 and 61–75 % increase in transformed plants, respectively. In contrast to control, DPPH, lipid peroxidation and DNA protection assay showed up to 37, 20 and 50 % enhancement in transformed plants, respectively. The extracts showed similar but significant enhancement behavior in hot plate analgesic and carrageenan-induced hind paw edema test. The transformed extracts showed 72.1 and 78.5 % increase for analgesic and anti-inflammatory activities, respectively. The transformants of *rol C* gene exhibited prominent antidepressant activity with 64–73 % increase compared with untransformed plants. In conclusion, the present work suggests that transformation with *rol C* gene can be used to generate lettuce with enhanced medicinally important properties, such as antioxidant, analgesic, anti-inflammatory and antidepressant potential.

## Introduction

Plant secondary metabolites are exclusive sources for food additives, flavors, pharmaceuticals and economically significant substances (Hussain et al. 2012). Plant cell cultures for extraction and multiplication of secondary metabolites can be produced characteristically under sterile conditions from explants, for example plant stems, meristems, leaves and roots (Hussain et al. 2012). Genetic transformation can manipulate biochemical pathways and produce transgenic crops in which nature, scope and range of existing natural products are modified to offer valuable agronomic and commercial post-harvest processing features (Karuppusamy 2009). Various methods are currently available for the genetic transformation of lettuce. The commonly used approach is *Agrobacterium*-mediated transformation. Previously, lettuce has been transformed with the gene-containing GDP-mannose pyrophosphorylase gene with 2.5-fold increase in vitamin C content (Wang et al. 2011). In another study, lettuce was transformed with synthetic folE and Atpsy gene, and a threefold increase in β-carotene content and 1.8-fold increase folic acid was measured (Li et al. 2012). These reports placed the basis for altering the metabolic pathways by transformation and earning the new lettuce varieties rich in pharmacological properties.

A number of phytochemicals including secondary metabolites and vitamins have antioxidant properties, which help in fending off the injury caused by stress-induced reactive oxygen species (El-beltagi and Mohamed 2013). Thus, the accumulation of antioxidants has a distinct adaptive advantage in plants. Typical phytochemicals with antioxidant properties are vitamins, such as α-tocopherol, ascorbic acid, carotenoids, phenolic and flavonoid compounds (Blokhina et al. 2003). These phytochemicals also have been reported to exert anticarcinogenic, anxiolytic and anti-inflammatory properties (Seeram et al. 2008; Harsha and Anilakumar 2013).

Lettuce has been used in the treatment of stomach problems to enhance appetite, to stimulate digestion and relieve inflammation (Sayyah et al. 2004). In addition, seed oil of lettuce has been reported for hypnotic, sedative, anticonvulsant and analgesic properties (Yakoot et al. 2011). Inflammation is normally characterized by pain, heat, swelling and redness while depression is common in chronic pain patients and it has been recommended that depression and pain share similar neurochemical mechanisms (Micó et al. 2006).

Antidepressants have been used as analgesic agent for several pain associated disorders like neuropathic and non-neuropathic pain as they possess intrinsic antinociceptive activity (McQuay et al. 1998). They inhibit the uptake of monoamines which leads to increase the amount of serotonin and noradrenaline in the synaptic cleft at both supraspinal and spinal levels that reinforce reducing pain inhibitory pathways (McQuay et al. 1998). Several reports proposed about the analgesic and anti-nociceptive activity of antidepressant drugs (reviewed by McCleane 2008), and they are generally identified for controlling chronic pain (Sindrup et al. 2005). Additionally, anti-inflammatory activity of clomipramine, fluoxetine and imipramine has been reported by using carrageenan-induced model (Abdel-Salam et al. 2003). In another investigation (Kast 2003) the potential for bupropion to decrease inflammation was evaluated. Although the exact mechanism is unknown but it can be suggested from the above reports that there is a link between all these activities. Considering the various activities reported in *Lactuca sativa,* this study was carried out to investigate the enhancement in its phytochemical, antioxidant, analgesic, anti-inflammatory and antidepressant potential by transformation with *rol C* gene.

## Materials and Methods

### Seed germination

The commercially available seeds of *Lactuca sativa* L. (cv. Grand Rapids) were surface sterilized with 70 % ethanol for 1 minute and sodium hypochlorite (10 %) for 30 seconds. Germination of sterilized seeds was carried out on ½ MS medium (Murashige and Skoog 1962).

### Bacterial strains and plasmids

*Agrobacterium tumefaciens* strain GV3101 containing plasmid pPCV002-CaMVC kindly provided by Dr. A. Spena, Max-Planck-Institute fur Zuchtungsforschung, 5000 Koin 30, FRG (Spena et al. 1987) were used for transformation. The T-DNA region of the plasmid pPCV002-CaMVC contained the coding sequence of *rol C* genes under the control of CaMV35S promoter. T-DNA of pPCV002-CaMVC also contained the *neomycin phosphotransferase* (*NPTII*) gene with nopaline synthase (NOS) promoter and NOS terminator sequences. *Agrobacterium tumefaciens* containing the plasmid pPCV002-CaMVC was grown overnight in Luria broth medium at 28 °C and 120 rpm in a shaking incubator. This bacterial culture was used for transformation purpose.

### Transformation and regeneration

One month old nodes and internode explants were used for transformation purpose. Explants were pre-cultured for 2 days on shooting media (0.1 mg/L NAA and 0.5 mg/L BAP), which were then given infection with bacterial strains containing the desired construct for 5 minutes. Explants were co-cultivated with bacteria for 2 days on shooting media supplemented with 200 µM acetosyringone. After 2 days of incubation in dark at 28 °C, explants were washed with antibiotics and placed on selection medium (0.1 mg/L NAA, 0.5 mg/L BAP, 50 mg/L kanamycin, 300 mg/L cefotaxime). Subculturing was done after every 14 days on selection media. Regeneration occurred within 1 month; three to four cycles of selection were given until complete transformed plants were regenerated on selection medium.

### Molecular analysis

Genomic DNA of transformed and untransformed (WT) plants was extracted by cetyltrimethyl ammonium bromide (CTAB) method (Clarke 2009). Plasmid was also isolated from GV3101 by alkaline lysis method. Polymerase chain reaction (PCR) was run using a programmed DNA thermal cycler (Biometra, USA). *rol C*: forward 5′-GAAGACGACCTGTGTTCTC-3′ and reverse primer 5′-CGTTCAAACGTTAGCCGATT-3′ and *NptII*: forward 5′-AAGATGGATTGCACGCAGGTC-3′ and reverse primer 5′-GAAGAACTCGTCAAGAAGGCG-3′ were used to amplify the respective genes. Previously reported PCR amplification conditions were followed (Kiani et al. 2012).

### Semiquantitative RT-PCR

Expression of *rol C* gene was analyzed by semiquantitative reverse transcriptase PCR. Total RNA was extracted from transformed and untransformed (WT) plants according to the reported procedure (Shirzadegan et al. 1991). DNAse treatment was given to the extracted RNA to confirm complete elimination of DNA. The purity and quantity of extracted RNA was checked by taking absorbance at 260 nm and 280 nm. Then 1–2 μg of RNA was reverse transcribed at 42 °C in a 20 μl reaction mixture containing 200 units of RevertAid M-MuLV (Moloney murine leukemia virus) reverse transcriptase (Thermo Scientific #K1622), according to the manufacturer’s instructions in the presence of RNAse inhibitor. PCR was carried out with *rol* C gene primers by using 1–2 μL of cDNA reaction mixture as template. The PCR products were evaluated on 1.5 % agarose gel containing ethidium bromide and photographed under UV. The band on agarose gel indicated the presence of mRNA and intensity of the bands indicated the quantity of mRNA levels.

### Phytochemical and antioxidant assays

#### Preparation of the plant extracts

Biomass extraction was carried out from untransformed and transformed aerial parts of *L. sativa* to carry out antioxidant assays. 1 g of dried powdered plant material was extracted with 20 mL methanol at room temperature in a sonication bath (Kerry Ultrasonic, UK) for 1 hour. The extracts were filtered using Whatman #1 filter paper and concentrated in vaco to generate the crude extracts. For experiments, extracts were prepared as 50 mg/mL in distilled water.

### Total phenolic contents (TPC)

TPC measurement was carried out according to the reported procedure (Moein et al. 2008) with some modifications. Briefly, 4 µL of each extract (50 mg/mL in distilled water) was transferred into each well of 96-well plate and 98 µL of Folin–Ciocalteu reagent diluted with distilled water (tenfold) was added. After mixing well, it was kept at 25 °C for 5 minute and then 98 µL 6 % Na_2_CO_3_ was added. Incubation of the resulting reaction mixture was carried out at 25 °C for 90 minutes and thereafter absorbance was measured at 725 nm. Here, distilled water was used as negative control and TPC were expressed as gallic acid equivalents.

### Total flavonoid contents (TFC)

Aluminum chloride colorimetric method was used for the total flavonoid content determination (Moein et al. 2008). 4 µL of each plant extract (50 mg/mL in distilled water) was mixed with 10 µL of 10 % AlCl_3_, 10 µL of 1 M potassium acetate and 176 µL of distilled water in each well of 96-well plate. Incubation of reaction mixture was carried out at 25 °C for 30 minutes. Absorbance was measured at 405 nm. Distilled water was used as negative control and TFC were expressed as quercetin equivalents.

### Total antioxidant capacity (TAC)

TAC was determined according to the previously reported method (Phatak and Hendre 2014). 4 µL (50 mg/mL in distilled water) of each plant extract was mixed with 196 µL of mixture containing 28 mM sodium phosphate, 4 mM ammonium molybdate and 0.6 M sulfuric acid. Then mixture was kept at 95 °C for 90 minute. After cooling reaction mixture at room temperature, absorbance was measured at 630 nm. Distilled water was used as negative control. TAC was expressed as ascorbic acid equivalent for each extract.

### Total reducing power (TRP)

TRP was measured according to the reported method (Moein et al. 2008). The TRP of the extracts was determined using 20 µL (50 mg/mL in distilled water) of extract mixed with 490 µL of 0.2 M of phosphate buffer and 490 µL of 1 % potassium ferricyanide. The reaction mixture was incubated for 20 minutes at 50 °C. Then 500 µL of 10 % trichloroacetic acid was added to the reaction mixture; the mixture was then centrifuged for 10 minutes at 3000 rpm. After taking 500 µL of the upper layer of the mixture in new eppendorf, 100 µL of 0.1 % ferric cyanide was added. Absorbance was measured at 630 nm. 100 µL of distilled water was used to blank the instrument. TRP of each sample was expressed as ascorbic acid equivalent.

### DPPH free radical scavenging assay

DPPH free radical scavenging activity of the plant extracts was determined according to the method reported by Ismail et al. (2015). DPPH (316 µM) was prepared in methanol. 2 µL of test sample was mixed with 98 µL of DPPH solution and then added in each well of 96-well plate. Incubation was carried out for 1 hour at 37 °C. Afterwards absorbance was measured at 515 nm. Assay was carried out at three different concentrations (1000, 500, 250 and 125 µg/mL) of each plant extract. Ascorbic acid was used as positive while distilled water was used as negative control. Percentage scavenging was calculated by the given formula and IC_50_ values were determined

$${\text{Percentage scavenging }}\left( \% \right) = \left[ {1 - \frac{\text{absorbance of extract}}{\text{absorbance of control}}} \right]\; \times \; 100.$$

### DNA protection assay

DNA protection activity of the extracts was measured in vitro (Ismail et al. 2015). The reaction mixture was prepared in PCR tube with total volume of 15 μL, having 3 μL pBR322 plasmid DNA (0.5 μg), 4 μL of 30 % H_2_O_2_, 3 μL of 2 mM FeSO_4_ and 5 μL of plant extracts at final concentration of 10, 100 and 1000 µg/mL. A positive control was used which contained pBR322 DNA treated with 2 mM FeSO_4_ + 30 % H_2_O_2_, untreated pBR322 DNA was used as negative control (P). Extract and pBR322 (C + P) was also used as a control to check the natural damaging or protective effect on DNA. Then the mixture was incubated for 1 hour at 37 °C. All reaction mixtures were subjected to 1 % agarose gel electrophoresis in 1× TBE buffer with 1 kb ladder (L). Gels were analyzed by scanning with Gel-Doc (BioRad) computer program and intensities of the bands were determined.

### Lipid peroxidation assay

The lipid peroxidation activity was determined according to the previously reported method (Gülen et al. 2008). For this purpose, lipid peroxidation was induced in liposomes prepared from egg lecithin through ultrasonic irradiation by adding 490 μL of 400 mM FeCl_3_ and 490 μL of 200 mM ascorbic acid. In this mixture, 20 μL of each plant extract was added with the final concentration of 1000, 500 and 250 µg/mL. Distilled water was used as negative control. The samples were incubated for 60 minutes at 37 °C. Then 1 mL of stopping solution comprising of 0.375 % (w/v) thiobarbituric acid, 1.5 % (v/v) trichloroacetic acid and 0.25 M HCl was added in the mixture to inhibit the reaction. These mixtures were placed in a boiling water bath for 15 minutes, cooled and centrifuged at 4000 rpm. Then 200 µL of the resulting solution was picked and added in each well of 96-well plate. Absorbance was measured at 532 nm. The percentage inhibition and IC_50_ value was calculated with table curve software

$${\text{Percentage inhibition }}\left( \% \right)\;{ = }\;\left[ {\frac{{{\text{Ac}} - {\text{As}}}}{\text{Ac}}} \right]\; \times \; 1 0 0 ,$$

where “Ac” means absorbance of control and “As” means absorbance of test sample.

### In vivo studies

#### Animals and treatment groups

Albino rats between 150 and 200 g of weight were used for the experiments. Rats were kept in standard aluminum cages and bred with water ad libitum and standard diet in the Primate facility of Faculty of Biological Sciences, Quaid-i-Azam University, Islamabad, Pakistan. The study design was approved by the Institutional Animal Ethics Committee and all provisions were carried to minimize animal suffering. Extracts and standard drugs were administered orally with the concentration of 500 and 10 mg/kg of the rat body weight, respectively to each group having five rats per treatment.

- Group-I: saline (10 ml/kg).
- Group-II: positive control (specific to model).
- Group-III: methanolic extract of untransformed wild type plant (WT).
- Group-IV: methanolic extract of *rol C1.*
- Group-V: methanolic extract of *rol C2.*
- Group-VI: methanolic extract of *rol C3.*

### Hot plate analgesic assay

Analgesic activity was determined by hot plate method first reported by Eddy and Leimbach (Eddy and Leimbach 1952), which is based on stimulation of pain by heat. Saline and aspirin (10 mg/kg) were used as negative and positive controls, respectively. Prior to oral dosage, rats were placed on the hot plate at 55 ± 2 °C to determine the jumping and paw licking response which was noted down as initial reaction time (IRT). After 30 minutes of dosage, each rat was placed on hot plate (55 ± 2 °C) and the basal response time was recorded by observing paw licking and jumping reaction (whichever seems first) was taken as final reaction time (FRT). The reaction time in seconds was recorded at different time intervals of 0.5, 1 and 2 hours after dosage with a cut-off period of 30 s. Percentage analgesic activity was calculated by the formula:

$${\text{Percentage analgesic activity = }}\left[ { \left( {\frac{{{\text{FRT}}\; - \;{\text{IRT}}}}{\text{IRT}}} \right)\; \times \; 1 0 0 } \right] .$$

### Carrageenan-induced hind paw edema test

Anti-inflammatory activity was determined by using carrageenan-induced hind paw edema test (Winter et al. 1962). Diclofenac potassium was used as positive control, whereas saline was used as negative control. After 1 hour of oral dosage of the plant extract, edema was induced by injecting 100 µL of carrageenan prepared in 1 % saline into the subplanter region of left hind paw. The paw volume was measured quickly before and after the carrageenan injection by using Plethysmometer (UGO Basile 7140), which served as the control readings of paw. Paw volume was measured after regular interval of 1 hour; readings were taken up to 4 hours. The percentage edema inhibition was determined by the formula:

$$\% \;{\text{inhibition = }}\left[ { \left( {\frac{{{\text{EDC}}\;{ - }\;{\text{EDT}}}}{\text{EDC}}} \right)\; \times \; 1 0 0 } \right] ,$$

where “EDC” is edema of control rats and “EDT” is edema of treatment rats.

### Forced swimming test

The anti-depressant activity of *L. sativa* was determined by forced swim test as described before (Slattery and Cryan 2012). One day before the experiment, rats were engaged in water containing vertical cylinder (18 cm diameter, 40 cm height and 15 cm; retained at 25 °C) separately and were forced to swim. In 5–6 minutes, the animal became steady and stayed motionless for almost 80 % of the time. After 15 minutes rats were evacuated, dried and placed back to their cages. This whole procedure was called pre-swimming. Fluoxetine HCl and saline were used as positive and negative controls, respectively. On the experiment day, after 30 minutes of dosage, rats were again placed in the water filled vertical cylinder and the camera was positioned to the side of the cylinder. Video recording was started and then the rats were placed in the cylinder. After 6 min, recording was stopped; the rats were removed from the cylinder, dried and placed back to their cages. When the experiment was completed, all videos were observed carefully (last 4 min of total recorded video) to estimate the total immobility time.

### Statistical analysis

The data obtained were statistically analyzed by one-way analysis of variance (ANOVA) and Turkey multiple comparison test. Results are represented as mean ± SD and *p* < 0.05 was considered to be significant. Percentage change of transformed lines (TransL) in comparison of untransformed plant (WT) was calculated by the following formula:

$${\text{Percentage change}}\;{ = }\;\left( {\frac{{{\text{TransL }} - {\text{ WT}}}}{\text{WT}}} \right) \times \; 1 0 0.$$

## Results

### Transformation and regeneration of lettuce plants

**Fig. 1:**
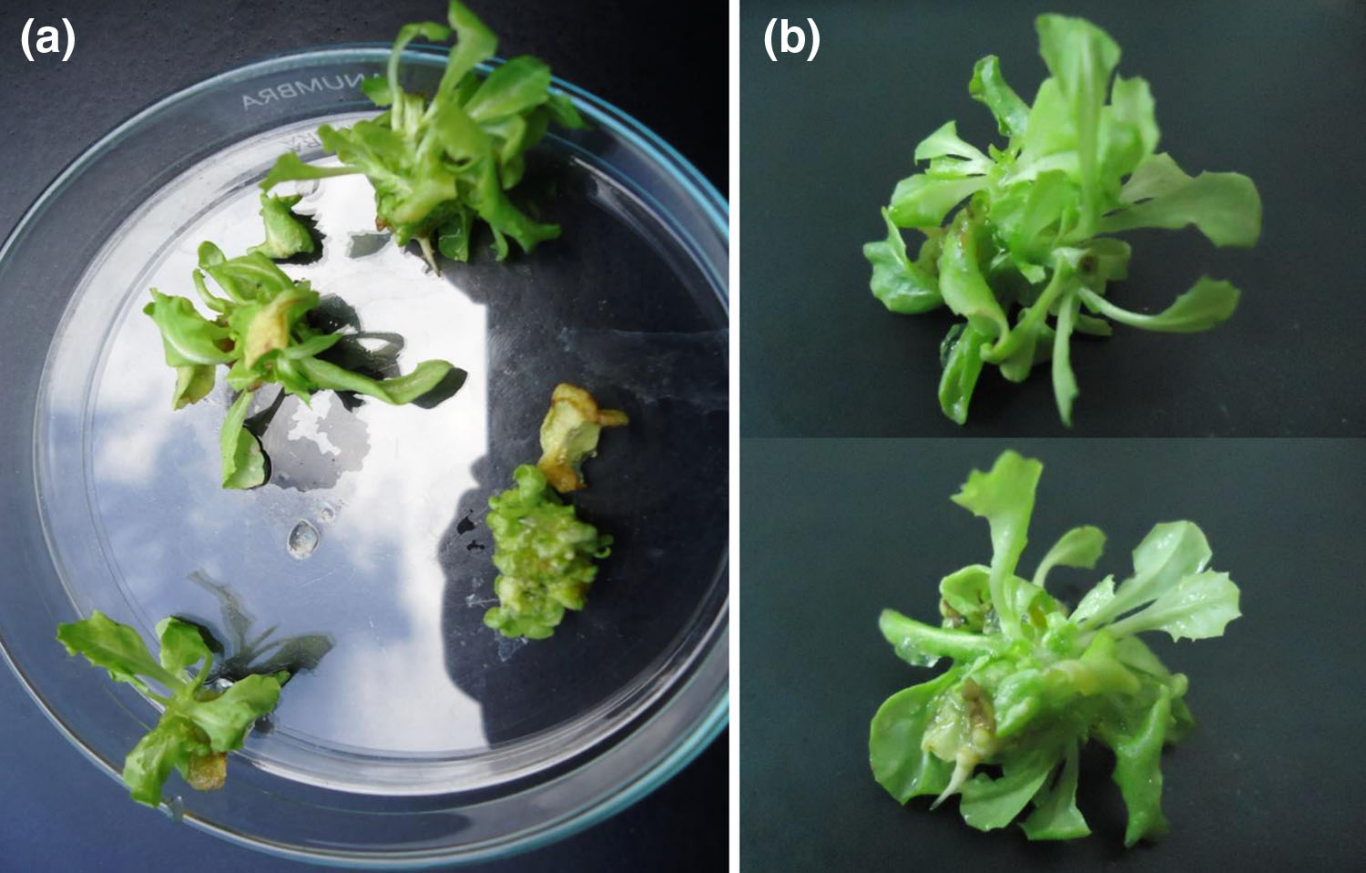
Plants of *Lactuca sativa.* **a** Untransformed plant and **b** *rol C* gene transformed plant

Lettuce was transformed with *Agrobacterium tumefaciens* GV3101 harbouring *rol C* gene. About 300 explants were transformed with the construct and the transformation efficiency was calculated as 65–75 %; but only three transgenic lines survived till maturity. In contrast to control, phenotypic alterations were observed in all transformed plants exhibiting reduction in inflorescence, stem heights, internodal lengths and leaf area (Fig. 1).

### Confirmation of integration and expression of *rol C* gene

**Fig. 2:**
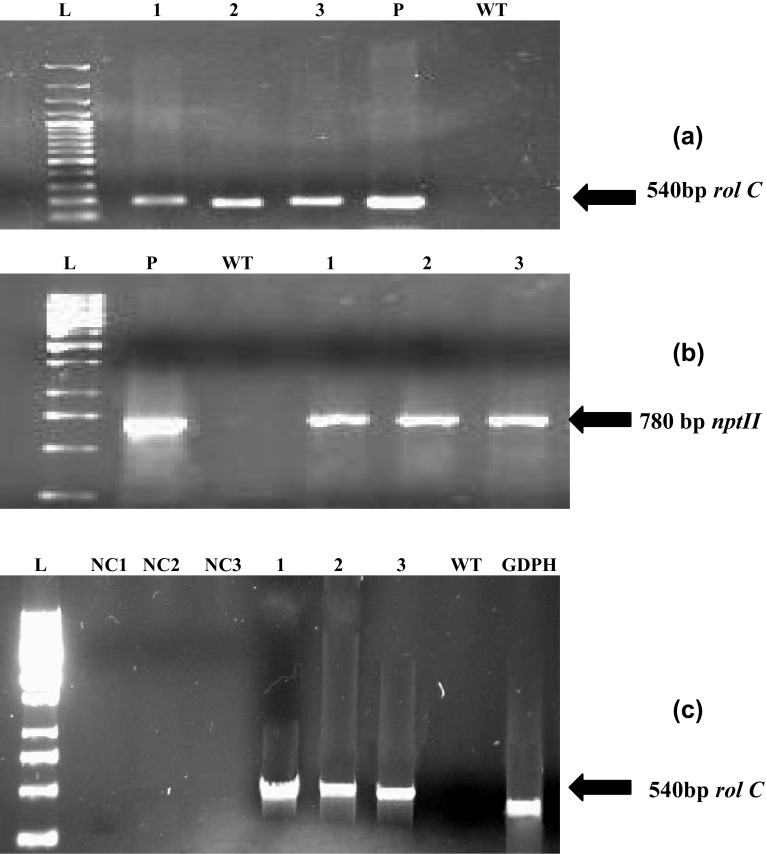
PCR amplified products of *Lactuca sativa.* **a** *rol C* gene, and **b** *nptII* gene are shown in the *figure*. Semiquantitative RT-PCR showing the relative expression of **c** *rol C* gene (transgenic lines 1–3). WT stands for untransformed plant of *Lactuca sativa* L. *Lane P* refers to the plasmid DNA and *lane L* corresponds to the 1 Kb ladder DNA (Fermentas). NT is reaction for each of the RNA samples without reverse transcription of respective gene and GADPH (496 bp) was used as positive control for the semiquantitative RT-PCR

The integration of *rol C* gene in the genome of *Lactuca sativa* was confirmed by performing PCR which showed the amplified products of 540 bp of *rol C* and 780 bp of *nptII* genes as shown in Fig. 2a, b. Plasmid DNA of GV3101-*rol C* was used as positive control which showed the respective amplified products for *rol C* and *nptII* genes. Wild type untransformed plants were used as negative control (WT) which showed no amplification. Semiquantitative reverse transcriptase PCR confirmed that the *rol C* gene was expressed in all transformed plants (Fig. 2c). For this expression analysis, RNA sample without reverse transcription was used as negative control (NC) and GADPH (provided with the kit) was used as positive control. The result showed that the expression of *rol C* gene in each line was not uniform. The transgenic line *rol C1* exhibited highest expression while *rol C2* showed moderate and *rol C3* showed lowest expression (Fig. 2c).

### Quantification of antioxidants

Total phenolic contents (TPC), total flavonoid contents (TFC), total antioxidant capacity (TAC) and total reducing power (TRP) of *rol C* gene transformed and untransformed plants of *Lactuca sativa* were determined and their comparative analysis was performed to find the correlation between them. TPC were expressed as equivalent of gallic acid per gram of plant dry weight (Table 1) and results showed 98.2, 71.7 and 53.5 % increase in transgenic line of *rol C1, rol C2* and *rol C3*, respectively compared with untransformed plants. TFC were expressed as equivalent of quercetin per gram of plant dry weight (Table 1) and results showed 58.3, 54.4 and 45.3 % enhancement for *rol C1, rol C2* and *rol C3*, respectively compared to untransformed plants. TAC and TRP both were expressed as equivalent of ascorbic acid per gram of plant dry weight (Table 1). The extracts of *rol C1* transformants showed 74.7 % increase, *rol C2* showed 71.2 % increase and *rol C3* exhibited 61.3 % increase in TAC as compared to untransformed plants. On the other hand results of TRP exhibited maximum increase in its contents with 118.4 % for *rol C1* transgenic line followed by 102.5 % for *rol C2* and 90.1 % for *rol C3* lines in comparison to untransformed plants. Overall results showed a significant positive correlation between TPC, TFC, TRP and TAC of all transgenic plants.

**Table 1:** Enhanced phytochemicals and antioxidant activities of *Lactuca sativa* extracts

Sr. no.	Assay	WT	*rol C1*	*rol C2*	*rol C3*
1	Total phenolic contents (TFC)	3.3 ± 0.3	6.5 ± 0.4**	5.7 ± 0.3**	5.1 ± 0.1**
2	Total flavonoid contents (TFC)	5.1 ± 0.1	8.2 ± 0.2**	8.0 ± 0.2**	7.5 ± 0.4**
3	Total antioxidant capacity (TAC)	3.7 ± 0.2	6.5 ± 0.1**	6.3 ± 0.1**	6.0 ± 0.2**
4	Total reducing power (TRP)	3.3 ± 0.1	6.4 ± 0.3**	5.9 ± 0.2**	5.6 ± 0.2**
5	DPPH scavenging assay	432.8 ± 15.1	273.6 ± 8.4**	306.1 ± 6.5**	366.8 ± 4.5**
6	Lipid peroxidation assay (LPA)	646.3 ± 15.7	515.9 ± 5.7**	520.2 ± 10.2**	546.1 ± 6.4**
7	DNA protection assay	+	+++	++	++

Each data represent mean ± SD (*n* = 3) of extract with * p < 0.05, ** *p* < 0.01 statistically significant. The values are expressed as milligram equivalents of gallic acid (TPC), quercetin (TFC) and ascorbic acid (TAC and TRP) per gram of plant dry weight, respectively. DPPH and LPA assays are expressed as IC_50_ (µg/ml). Ascorbic acid (IC_50_ 9.52 µg/mL) and vitamin E (IC_50_ 8.16 µg/mL) were used as positive controls for DPPH and LPA assays, respectively. In DNA protection assay “+” represent the slight, “++” moderate and “+++” good protection

### Measurement of antioxidant activities

**Fig. 3:**
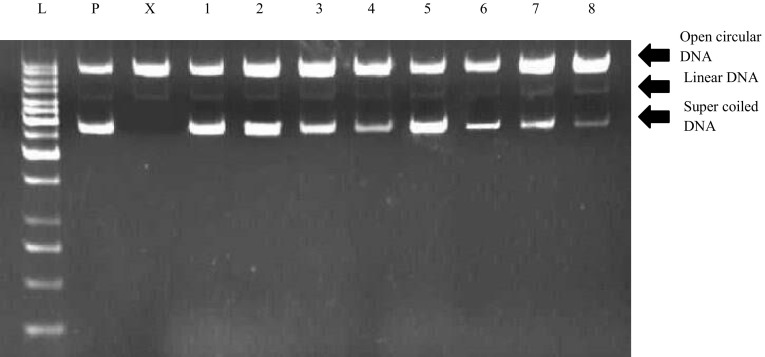
Results of DNA protection assay. Untrasformed extracts and representative transgenic line *C*1 of *rol C* gene showing DNA protective effect on pBR322 plasmid DNA [*lane# L* DNA ladder (1 Kb), *P* pBR322 plasmid, *X* pBR322 plasmid treated with FeSO_4_ and H_2_O_2_ (positive control), *1* pBR322 plasmid + 1000 µg/mL of *rol C1* line extract; control for the pro-oxidant effect of the extract on DNA, *2* plasmid + 1000 µg/mL of *rol C1* line extract + FeSO_4_ + H_2_O_2_, *3* plasmid + 100 µg/mL of *rol C1* line extract + FeSO_4_ + H_2_O_2_, *4* plasmid + 10 µg/mL of *rol C1* line extract + FeSO_4_ + H_2_O_2_, *5* pBR322 plasmid + 1000 µg/mL of untransformed extract; control for the pro-oxidant effect of the plant extract on DNA, *6* plasmid + 1000 µg/mL of untransformed extract + FeSO_4_ + H_2_O_2_, *7* plasmid + 100 µg/mL of untransformed extract + FeSO_4_ + H_2_O_2_, *8* plasmid + 10 µg/mL of untransformed extract + FeSO_4_ + H_2_O_2_

DPPH free radical scavenging assay is a spectrophotometric method for the screening of antioxidant potential of plant extracts. In this study, comparative estimation of antioxidant activity was carried out for *rol C* transgenic plants and untransformed wild type plants. Ascorbic acid (IC_50_ 0.01 mg/mL) and distilled water served as positive control and blank, respectively. Results showed significant enhancement in antioxidant activity for all transgenic lines than that of untransformed plants (Table 1). The extracts of *rol C1* transgenic plants showed the highest DPPH radical scavenging potential with an IC_50_ value of 0.27 mg/mL which is 36.4 % increase compared to untransformed plants with an IC_50_ of 0.43 mg/mL. The extracts of *rol C2* and *rol C3* transformants showed 28.8 and 14.7 % increase as compared to extracts of untransformed plants (Table 1). DNA protection assay was performed to further evaluate the scavenging property of transformed and untransformed plants. Quantification of bands obtained on 1 % agarose gel showed 50 % more dense band in transgenic lines in comparison of untransformed plants, which represents its enhancement (Fig. 3; Table 1). All three transgenic lines exhibited significantly high protection in concentrations dependent manner. Additionally, transformed extracts were tested for the inhibition of lipid peroxidation by the thiobarbituric acid assay. Vitamin E was used as a positive control (IC_50_ 0.003 mg/mL) and distilled water served as blank. The transgenic lines *rol C1*, *rol C2, rol C3* showed enhanced activities with IC_50_ 0.51, 0.52 and 0.54 mg/mL, respectively while untransformed plant exhibited IC_50_ value of 0.65 mg/mL (Table 1). Overall, about 20 % increase in activity was observed in transformed plants as compared to untransformed plants.

### Analgesic assay

**Fig. 4:**
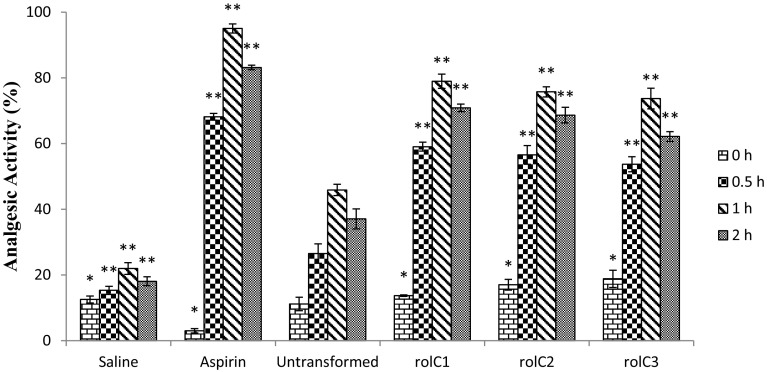
Results of analgesic assay of *Lactuca sativa* extracts. Transformed lines of *rol C* (1–3) and untransformed (WT). Each group (*n* = 5) is expressed in mean ± SD. *p < 0.05, **p < 0.01 statistically significant

The extracts of *Lactuca sativa* transformed with *rol C* gene showed significant increase in analgesic activity in rats. Latency period of each rat was determined and percentage inhibition was calculated which showed gradual increase in activity for 0.5 and 1 hour after oral dosage followed by decrease in activity at 2 hours (Fig. 4). Aspirin was used as positive control (95 % inhibition) and saline was used as negative control (22 % inhibition). The results were compared with untransformed wild type extract (WT) which showed 45.9 % analgesic activity. In contrast to control, the highest activity was calculated by *rol C1* with 79.1 % reduction in analgesia which is about 72.1 % higher than that of untransformed plants. In case of *rol C2* and *rol C3* transgenic lines, 65.1 and 60.7 % increase in analgesic activities were recorded, respectively.

### Anti-inflammatory assay

**Fig. 5:**
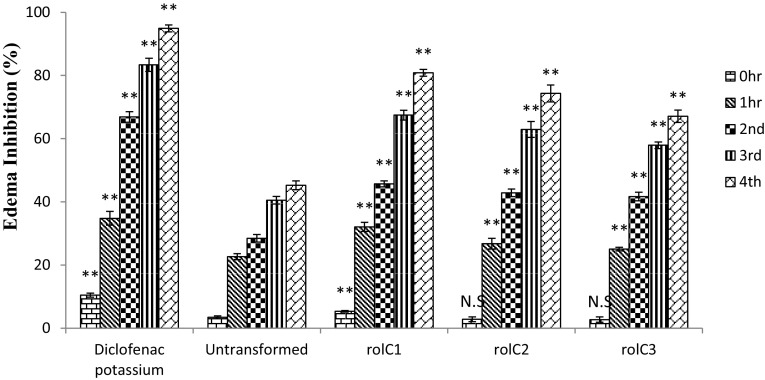
Results of anti-inflamatory assay of *Lactuca sativa* extracts. Transformed plants of *rol C* (1–3) and untransformed (WT). Each group (*n* = 5) is expressed in mean ± SD. *p < 0.05, **p < 0.01 statistically significant

The anti-inflammatory activity of the transformed and untransformed extracts was evaluated by carrageenan-induced hind paw edema test in rats. The edema volume was measured by Plethysmometer (UGO Basile 7140) and the percentage inhibition in edema was calculated after the injection of carrageenan at 1, 2, 3 and 4 hours (Fig. 5). All the transgenic lines showed time dependent increase in activity, maximum at 4 h. Diclofenac potassium was used as positive control (95 % inhibition) and saline served as negative control (0 % inhibition). The extracts of *rol C1* line showed maximum activity with 80.8 % edema inhibition which is about 78.5 % higher activity than that of untransformed control plants (45.3 % inhibition). The extracts of transgenic lines *rol C2* and *rol C3* showed 64.1 and 48.1 % increase in anti-inflammatory activities, respectively.

### Antidepressant assay

**Fig. 6:**
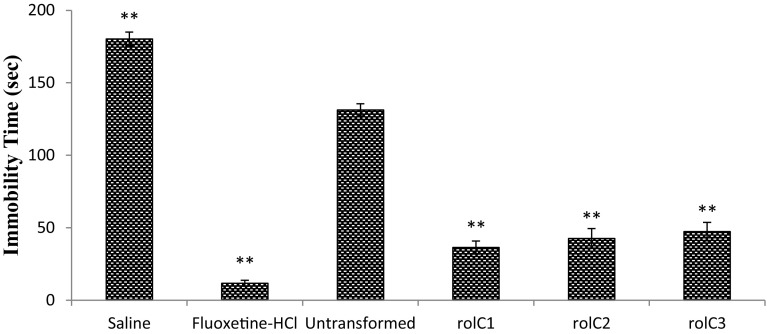
Results of anti-depressant assay of *Lactuca sativa* extracts. Transformed lines of *rol C* (1–3) and untransformed (WT). Each group (*n* = 5) is expressed in mean ± SD. *p < 0.05, **p < 0.01 statistically significant

Antidepressant potential of transformed and untransformed lettuce extracts were investigated in rats by forced swimming test. The transgenic line *rol C1* exhibited highest antidepressant activity with the immobility time of 36 ± 3 s as compared with untransformed extracts (131 ± 5 s; Fig. 6). Fluoxetine-HCl and saline were used as positive and negative controls with the immobility time of 11 ± 1 and 180 ± 3 s, respectively. Overall, the *rol C1*, *rol C2* and *rol C3* transgenic lines displayed antidepressant activities with 72.5, 67.2 and 64.1 % enhancement, respectively, as compared to untransformed control plants.

## Discussion

The use of the medicinal plants for curing disease has been documented in history of almost all civilizations (Gousia et al. 2013). One such plant *Lactuca sativa* is famous for its medicinal properties, such as antioxidant, anti-inflammatory, analgesic and sedative-hypnotic effects (Fu et al. 2012). Complete profile of pharmacological activities of lettuce as well as molecular modes of actions can further elucidate the full potential of this important crop. The aim of this study was to enhance the production of secondary metabolites in *Lactuca sativa* (cv. Grand Rapids). To achieve this, lettuce was transformed with *rol C* gene by using *Agrobacterium tumefaciens*. PCR analysis confirmed the presence of transgene and semiquantitative reverse transcriptase PCR was performed to study the expression which measures the mRNA level specific to DNA level. It has been seen that the *rol* genes of *Agrobacterium rhizogenes* played an essential role in the activation of secondary metabolites in transformed plants. We also report some morphological differences that existed among transgenic and non-transgenic plants. Such features were similar in all transformed plants which show that these changes are associated with transformation and not due to somaclonal variation. Previously, similar type of growth reduction was observed in *Lactuca sativa* L. (cv. Lake Nyah) when transformed with *Agrobacterium rhizogenes rol AB* genes (Curtis et al. 1996).

It is well known that consumption of fruits and vegetables is linked with reduced risk of diseases. This is due to their high levels of phytochemicals that prevent diseases related to oxidative stress in the human body. Understanding the distribution of phytochemical profile in vegetables and fruits is of primary importance. So, all the transformed and untransformed plants were evaluated for their TPF, TFC, TRP, TAC and antioxidant activity by using DPPH, DNA protection and lipid peroxidation assays. A significant positive correlation was found among all enhanced phytochemicals and antioxidant activities in transformed plants. The phytochemical studies on lettuce have already reported that it contains a number of flavonoids (Caldwell 2003). Synergistically, these dietary antioxidants provide bioactive mechanisms to reduce free radical induced oxidative stress. The DNA damage and oxidative stress conditions are the results of free radical production initiated by imbalance state of antioxidants and oxidants. In our results, transformed plants showed enhanced DNA protection response due to the effect of *rol C* gene as compared with previous report (Harsha et al. 2013). The protective effect of extracts on DNA may be attributed to the presence of phenolics and flavonoids. These results are supported by the previous findings (Bulgakov 2008) which states that suppressed levels of reactive oxygen species (ROS) is as a consequence of higher antioxidant activity in *rol C* transformed cells. Likewise, light stimulated elevated levels of ROS were decreased in the *rol C* transformed cells as compared to the controls (Bulgakov 2008). Enhanced antioxidant activity by transformation with *rol* genes may improve the plant defence response by suppressing the oxidative damages.

Transgenic lines of *rol C* gene were investigated for their analgesic, anti-inflammatory and anti-depressant activities in rats. Hot plate assay is one of the most suitable and easy methods for the investigation of centrally acting analgesic involving spinal reflexes (Sharma et al. 2013) while inflammation process is biphasic regulating process induced by carrageenan. The initial phase (1–2 h) of the inflammation is caused by the release of serotonin ad histamine while the final phase (3–4 h) is considered by the peak volume of hind paw (Khan et al. 2009). Analgesic and anti-inflammatory studies showed significant enhancement with a strong positive correlation representing that they might share a similar mechanism at some level. Recently, the presence of flavonoids has been studied in *Lactuca* species and these flavonoids have been reported to halt prostaglandin synthetase (Hugar et al. 2010). Since prostaglandins are involved in pain perception as well as released in the inflammation response, it could be proposed that limited accessibility of prostaglandins by flavonoids might be involved in its analgesic effect and reduced inflammation. We also investigated the anti-depressant effect of *L. sativa* via forced swimming test that represents the pharmacological model and produces a state similar to human depression (Glory et al. 2014). In literature, it has been reported that treatment with antidepressants reduces the oxidative stress related to depressive disorder and flavonoids contain antioxidant property which is demonstrated experimentally by the rise of the plasma antioxidant status (Pietta 2000). Although, it has been identified that plants containing flavonoids exhibit antidepressant activity (Moallem et al. 2007), antidepressant effect of various flavonoids and phenolics contained in *Lactuca sativa* needs to be further explored.

## Conclusion

The results of current study on *Lactuca sativa* (CV. Grand Rapid) transformed with *rol C* gene showed significant enhancement in secondary metabolites compared with untransformed wild type plants. It is interesting to note that three transgenic lines showed a fixed pattern in all the activities. All three transgenic lines showed increase in phytochemicals and antioxidants and enhanced analgesic, anti-inflammatory and anti-depressant activities. In short, it can be concluded that *rol* genes can be potentially used to enhance antioxidant and medicinal properties.
